# *InVitro* and *Ex Vivo* Biocompatibility, Biomolecular Interactions, and Characterization of Graphene Quantum Dots and Its Glutathione-Modified Variant for Qualitative Cell Imaging

**DOI:** 10.1021/acsomega.4c10014

**Published:** 2025-04-15

**Authors:** Marlin Pedrozo-Peñafiel, Luis Gutierrez-Beleño, Cesar A. D. Mendoza, Fernando L. Freire-Júnior, Mauro A. Lima, Tamara Teixeira, Fillipe V. Rocha, Gabriela V. S. Zolin, Saulo S. Garrido, Ana B. Lazzarini, Adelino V. G. Netto, Felipe F. Haddad, Emilio E. João, Jean L. Santos, Cauê B. Scarim, Renan L. Farias, Ricardo Q. Aucélio

**Affiliations:** a Dept. of Chemistry, Pontifical Catholic University of Rio de Janeiro (PUC-Rio), Rio de Janeiro, RJ 22451-900, Brazil; b Dept. of Electric Engineering, Rio de Janeiro State University (UERJ), Rio de Janeiro, RJ 20550-900, Brazil; c Dept. of Physics, Pontifical Catholic University of Rio de Janeiro (PUC-Rio), Rio de Janeiro, RJ 22451-900, Brazil; d Dept. of Chemistry, Federal University of Sao Carlos (UFSCar), Sao Caerlos, SP 13565-905, Brazil; e Dept. of Biochemistry and Organic Chemistry, Sao Paulo State University (Unesp), Araraquara, SP 14800-060, Brazil; f Dept. of Analytical, Physicochemical and Inorganic Chemistry, Sao Paulo State University (Unesp), Araraquara, SP 14800-060, Brazil; g School of Pharmaceutical Sciences, Sao Paulo State University (Unesp), Araraquara, SP 14800-903, Brazil

## Abstract

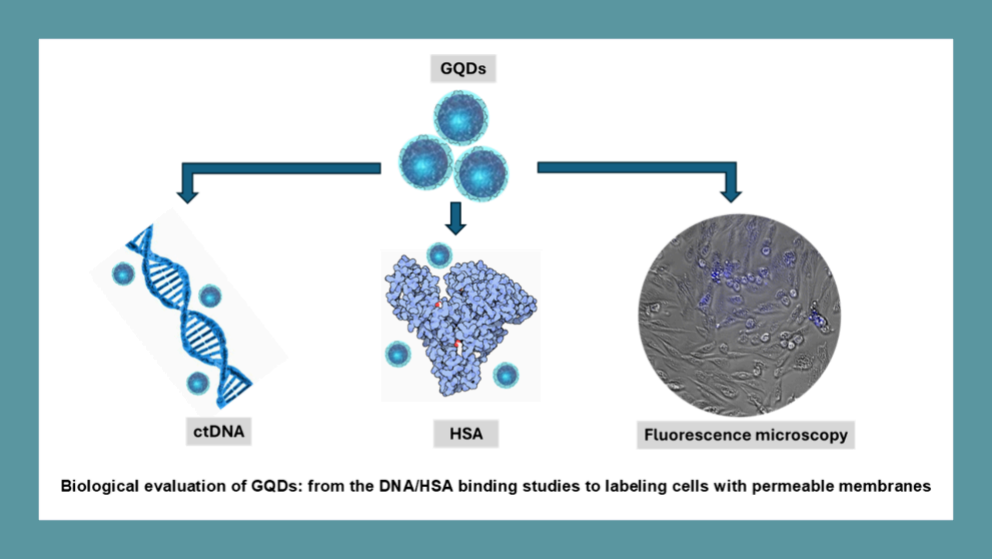

This study explores the biological effects and imaging potential of graphene quantum dots (GQDs) and their nitrogen-doped, glutathione-modified form (GQDs-GSH) in human cell models. We evaluated the cell viability, hemolysis potential, and irritation effects of these nanomaterials, showing that both GQDs and GQDs-GSH maintained 100% cell viability in the range of 16.25–260 μg mL^–1^, caused minimal RBC hemolysis (<9%), and presented low irritation scores in HET-CAM assays. Interaction studies revealed distinct binding behaviors with biomolecules: GQDs used a static quenching mechanism with human serum albumin (HSA), while GQDs-GSH exhibited a mixed static and dynamic interaction. Additionally, GQDs-GSH exhibited a stronger affinity for calf thymus DNA (ctDNA) without disturbing the DNA secondary structure. Fluorescence microscopy-based apoptotic cell staining assays indicated the adherence potential of both GQDs and GQDs-GSH. GQDs tended to accumulate more prominently in regions associated with apoptotic bodies, whereas GQDs-GSH tended to localize on the membranes of spheroid-shaped cells.

## Introduction

A subset of quantum dots (QDs),^1^ known as graphene quantum dots (GQDs), consists of a monolayer or a few stacked graphene sheets within a size range from 10 to 100 nm. GQDs exhibit quantum confinement, edge effects, and a high surface area-to-volume ratio. Because of quantum confinement, GQDs have size-dependent properties, behaving differently based on their volume, morphology, and composition. GQDs offer notable characteristics such as photostability and intense luminescence, dependent on particle size, morphology, and composition. They are also compatible with aqueous solutions and are reported to be biocompatible.^2,3^

Various methods exist for GQDs production, including top-down approaches that involve the reduction of large graphene sheets through electrochemical oxidation, ultrasonic-assisted synthesis, acid exfoliation, and ablation through beam irradiation.^3−9^ Conversely, the bottom-up procedures start from small molecules and are based on pyrolysis, microwave-assisted processes, hydrothermal exfoliation, and stepwise organic synthesis.^3,8,9^ Natural precursors are employed to synthesize high-quality GQDs using green strategies. Citric acid, ascorbic acid, and glucose are highlighted as carbon sources due to their widespread occurrence, low cost, and biocompatibility.^4,10,11^

The scientific exploration of GQDs tailored for the specific labeling of apoptotic bodies, as opposed to viable cells, is essential for biomedicine.^5,10,12^ The ability to discern apoptotic cells from their healthy counterparts holds importance for precision diagnostics and targeted treatments, spanning diverse medical domains such as oncology, neurology, and immunology.^13^ Studies conducted by Roy et al. (2014)^14^ showcase the effectiveness of plant leaf-derived GQDs modified with annexin V antibody (AbA5) in selectively labeling apoptotic cells within the zebrafish model. The notable outcome is the zebrafish’s capacity to emit bright red photoluminescence in the presence of apoptotic cells, emphasizing the potential of GQDs as a valuable tool for *in vivo* imaging.

However, this potential intersects with a broader challenge of understanding toxicity and establishing regulatory frameworks to oversee nanomaterials. Furthermore, the biological interaction mechanisms of GQDs with essential biomolecules, such as DNA and serum proteins, necessitate extensive research for comprehensive understanding. Lin et al. (2019)^15^ revealed significant effects on fetal length in pregnant mice exposed to a high GQD dosage (30 mg kg^–1^) at 8.5 days of gestation. Interestingly, the second-generation female mice exposed to this treatment showed average growth, reached sexual maturity, and successfully gave birth to healthy offspring. Senel et al. (2019) demonstrated a dose-dependent modulation of cell viability, as well as antimicrobial and antioxidant activities for *N*-doped GQDs. Notably, the results underscored the interaction between *N*-doped GQDs and DNA structure through intercalation and electrostatic binding. Furthermore, the authors proposed using GQD-based siRNA formulations to mitigate the systemic toxicities associated with chemotherapy in anticancer treatments. The investigation revealed that high GQDs concentrations can reduce cell viability by damaging DNA in cancer cells, while at lower concentrations, GQDs offer bioimaging capabilities.^5^

Herein, as part of our ongoing research on GQD-based nanomaterials for biosensing applications, we delve into a comprehensive examination of the biological effects of both GQDs and one N-doped GQDs produced by the fusion of citric acid and a mixture of citric acid and l-glutathione (GQDs-GSH), respectively, followed by hydro-exfoliation. This work assesses the impact of these nanomaterials on tissue cell viability, hemolysis, and binding-ligand interactions with HSA/DNA, employing spectrophotometry, fluorescence, and circular dichroism. While literature covers various GQDs applications for quantifying this biomolecule, we expand their scope by demonstrating that HSA serves as a transport protein for these nanomaterials, a previously unexplored aspect. Moreover, this is the first study to evaluate GQDs using the Hen’s Egg Chorioallantoic Membrane (HET-CAM) assay, providing novel insights into their hemorrhagic and irritant profiles and thereby enhancing the understanding of biocompatibility.

## Experimental Section

### Chemicals

Ultrapure water (18.2 MΩ cm) was obtained from the Milli-Q gradient A10 ultrapurifier (Millipore, USA). *Calf thymus* DNA (ctDNA) and Human serum albumin (HSA) were obtained from Sigma-Aldrich (USA). Dialysis membranes, which retained a molecular weight of 3.5 kDa, were obtained from Spectrum Laboratories Inc. (USA). Sodium hydroxide (98.0% m/m), citric acid, and *L*-glutathione were obtained from Merck (Germany). The 3-(4,5-imethylthiazol-2-yl)-2,5-diphenyltetrazolium bromide (MTT) was obtained from Sigma-Aldrich (USA).

### Production and Characterization of GQDs

GQDs were produced using an eco-friendly method (without high temperatures or high-pressure systems) using the fusion and hydro-exfoliation of organic precursors.^11^ First, 1.0 g of citric acid was placed into a 5 mL beaker and heated to about 240 °C using a heating plate. As citric acid melted (with color changing from colorless to pale-yellow and then to brown) within 2–5 min, 100 mL of ultrapure water at room temperature was poured over the hot molten material, resulting in a clear pale-yellow aqueous dispersion that was further dialyzed, for 24 h, to obtain the so-called GQDs aqueous original dispersion. Alternatively, this same procedure was made using a mixture of 1.0 g of citric acid and 0.3 g of *L*-glutathione (GSH), used as a chemical modifier, aiming for functionalization of produced carbon nanostructures, leading to the so-called GQDs-GSH original dispersion.

Photoluminescence measurements were made on a model LS 55 luminescence spectrophotometer (PerkinElmer) (1000 nm min^–1^ scan rate, 10.0 nm spectral bandpass, and 1 cm optical path length quartz cuvettes). Total carbon measurements were made using a carbon analyzer model TOC-VCPN (Shimadzu, Japan). Dynamic light scattering (DLS) and zeta-potential measurements were made on a nanoparticle analyzer model SZ-100 (Horiba, Japan). Zeta potential analyses were conducted using an acrylic electrochemical cell with a flat carbon electrode that has a thickness of 6 mm. DLS measurements were obtained using glass cuvettes with a 1 cm optical path length. The pH measurements were made on a pH meter model mPA 210 (Tecnopon, Brazil) with a glass membrane electrode conjugated with an Ag*|*AgCl (KCl_(sat)_) reference electrode. Sonication was performed using an ultrasonic bath (9 L, NSC 2800 model, Unique, Brazil).

Raman spectroscopy was performed using an NT-MDT Raman NTEGRA equipped with a solid-state laser with a 2.62 eV (473 nm) line with a 1.0 μm spot size with an acquisition time of 30 s, using filters employed to avoid overheating of the sample, with the measurement at room temperature and atmospheric pressure. The specimens were prepared on silicon dioxide (SiO_2_) substrates with an approximate thickness of 285 nm. Each drop of dispersion with a volume of about 2.5 μL was placed sequentially onto the substrate. After each drop, the samples were dried on a hot plate at 70 °C until 50 μL of the sample had been applied.

The infrared spectra were obtained on a Bruker ALPHA II FTIR spectrometer using an Eco-ATR QuickSnap Sampling Module (ZnSe crystal).

STEM images were carried out with a field emission scanning electron microscope (FEG-SEM, JSM6701F, from JEOL) operating at 30 kV. Images were processed using Image-J free software to obtain the mean diameter of GQDs and GQDs-GSH. All grids used were thoroughly checked to prevent any artifacts. Samples were prepared on grids of type lacey carbon film on 300 mesh copper using a drop (2.5 μL) of sample dispersion, letting it dry under normal pressure and room temperature.

### MTT Cell Viability Assay

The cells A549 (lung cancer, ATCC–CCL-185), MRC-5 (nontumor lung fibroblast, ATCC–CCL-171), and DU-145 (prostate cancer, ATCC–HTB-81) were maintained in DMEM (Dulbecco’s Modified Eagle’s Medium) supplemented with 10% heat-inactivated FBS. The cells were placed in cell culture flasks (Corning) at 310 K in a humidified 5% CO_2_ atmosphere. Cell counting was done using the Trypan Blue dye exclusion method.^16,17^ The cells were added in 150 μL (1.5 × 10^4^ cells) of the medium into each of the wells of a 96-well culture plate to be incubated for 24 h. Then, 50.0 μL of the original GQDs or GQDs-GSH dispersion was added to the wells at various concentrations, resulting in nanomaterial concentrations in the wells of 16.25, 32.50, 65.0, 130, and 260 μg mL^–1^ (n = 3). The effect of the GDQs on the cells was determined in 48 h. After the incubation, 50 μL of 1 mg L^–1^ MTT (3-(4,5-imethylthiazol-2-yl)-2,5-diphenyltetrazolium bromide) solution was added to the wells, and the plates were incubated for 4 h. The resulting extinctions were measured at 540 nm in a BioTek Epoch microplate spectrophotometer reader.

### Red Blood Cell (RBC) Hemolytic Profile

The hemolytic activity was evaluated through the protocol described by Farias et al. (2021)^18^ with minor modifications. Accordingly, human red blood cells (RBC) were obtained from the fresh blood (type O+) of a healthy volunteer (CAAE n° 61293316.4.0000.5426). The collected blood was 3-fold washed with a saline phosphate buffer (PBS) 0.01 mol L^–1^ and centrifuged to separate RBC and plasma. The final erythrocyte solution equals 10 μL of washed RCB in 990 μL of PBS buffer. Afterward, the nanomaterials were prepared in Eppendorf tubes with 13, 26, 52, and 104 μg mL^–1^. Triton X-100 at 1% (v/v) was used as a positive control (100% of hemolysis), while PBS buffer 0.01 mol L^–1^ was used as a negative control (0% of hemolysis). Aliquots of 100 μL of the erythrocyte suspension and 100 μL of each compound were incubated at 310 K for 1 h. The samples were centrifuged at 3000×g for 3 min, and 100 μL of each mixture was added to specific wells of a 96-well cell culture plate. For each mixture, the optical extinction was measured by using an Epoch Biotek microplate reader at 540 nm. The % hemolysis caused by the nanomaterials was determined according to eq 1
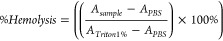
1where the *A*_*sample*_ regards the extinction obtained in the microplate measurements. *A*_*Triton1%*_ and *A*_*PBS*_ represent the extinction values obtained in the positive or negative controls.

### Hen’s Egg Chorioallantoic Membrane Assay (HET-CAM)

Fertilized Hen’s eggs (Novogen Brown) were incubated horizontally and placed in an automatic incubator for 6 days under controlled temperature (310.65 ± 0.65 K) and relative humidity (60 ± 5%). To ensure fertility, the nonviable eggs were discarded on the third day. The eggs were replaced in the incubator with the large end upward. On the ninth day, preparations for the assay commenced with removing the shell segment above the air cell. Subsequently, the membrane was moistened with a 0.9% NaCl solution prior to its meticulous removal. The experiment involved four groups: one standard group comprising three eggs (n = 3) that were treated with 0.1 mol L^–1^ NaOH; one control group consisting of three eggs (n = 3) that were treated with a 0.9% saline solution (NaCl); and two sample groups, each consisting of three eggs (n = 3), where one group was treated with GQDs and the other with GQDs-GSH. Two different volumes (100 and 300 μL) of the original dispersions of GQDs or GQDs-GSH were directly added to the CAM, and the effect was observed for 300 s. The irritation score (IS) included hemorrhage, vascular lysis, and coagulation times (in seconds) and was reported according to eq 2.
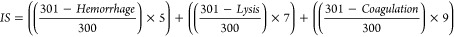
2

The semiquantitative HET-CAM score^19,20^ was measured and the survival rate was monitored for 10 min after each GQDs dosing.

### Human Serum Albumin (HSA) Binding Studies with GQDs/GQDs-GSH

Binding studies were made by measuring the photoluminescence of HSA^18,21,22^ within the emission range between 290 and 440 nm, with a maximum at 347 nm, under excitation at 280 nm. The HSA fluorescence quenching was monitored upon increasing additions of GQDs and GQDs-GSH. The stock solution of the HSA (1.0 × 10^–3^ mol L^–1^) was prepared in Tris-HCl buffer (pH 7.4) and diluted to 4.0 × 10^–7^ mol L^–1^ to perform experiments. Fluorescence (F) measured from HSA solution in the presence of GQDs or GQDs-GSH was normalized by the signal measured from HSA solution in the absence of the nanomaterials (F_0_) to establish a mathematical relationship between normalized signal (F_0_/F) and quencher concentration. In this manner, the sensitivity is the quenching constant (*K*), as seen in eq 3, where *K* can be solely from static quenching (*K*_*S*_) or a mixed process consisting of static and dynamic quenching (*K*_*S*_ and *K*_*D*_).
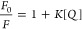
3

The binding constant values and the number of binding sites (*n*) were obtained using eq eq 4, where *K*_*b*_ and *n* are the binding constant and the number of binding sites, respectively.
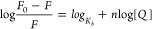
4

### DNA Binding Studies with GQDs/GQDs-GSH

#### UV–Visible Extinction Spectrophotometric Titration

Binding studies between *Calf thymus* DNA (ctDNA) and GQDs/GQDs-GSH were evaluated through absorption UV–visible spectroscopy. The ctDNA stock solution was prepared in Tris-HCl buffer (1.0 × 10^–3^ mol L^–1^; pH 7.4), and its concentrations were determined spectrophotometrically using the Beer–Lambert law (ε = 6600 L mol^–1^ cm^–1^) as 1.46 × 10^–4^ mol L^–1^. The affinity of the nanomaterials for ctDNA was assessed by determining the intrinsic binding constant (K_b_). This evaluation was based on the maximum extinction observed at a specific wavelength for GQDs at a concentration of 149.5 mg L^–1^ and GQDs-GSH at 102.9 mg L^–1^, alongside the incremental addition of ctDNA concentrations ranging from 2.0×10^–6^ to 4.4×10^–5^ mol L^–1^. The Benesi–Hildebrand equation was used according to eq 5, where A and A_0_ are the extinctions of the GQDs or GQDs-GSH in the presence and absence of ctDNA, respectively. The ε_G_ and ε_H-G_ are the molar absorptivity coefficients of the free GQDs or GQDs-GSH and GQDs/DNA, GQDs-GSH/DNA adduct. The *K*_*b*_ is given by intercept to slope ratio of a 1/(A-A_0_) vs 1/[DNA] plots.
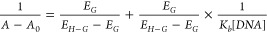
5

#### Circular Dichroism (CD)

A solution of ctDNA (16.5 μg mL^–1^) dissolved in Tris-HCl buffer (1.0×10^–3^ mol L^–1^; pH 7.4) and increasing concentrations of GQDs (0 – 6.50 μg mL^–1^) or GQDs-GSH (0 – 67.2 μg mL^–1^) were incubated for 24 h at 310 K. Then, CD spectra were collected at 298 K in a JASCO J-815 spectropolarimeter between 235 and 320 nm using the accumulation of 5 scans and a scan speed of 100 nm min^–1^.

#### Fluorescence-Labeled Cell Imaging

The cells of MRC-5 (1.0×10^4^ cells) were seeded in a microplate with 96 wells; after 24 h of incubation, 1 μL of two solutions of cisplatin (6.25×10^–6^ and 12.5×10^–6^ mol L^–1^) were added in the wells (two replicates used). Sequentially, the GQDs and GQDs-GSH (100 μg mL^–1^) were added, totaling 200 μL, and the microplate was incubated for 48 h. After this, the medium was removed, and the cells were fixed with methanol and stained with PI (propidium iodide) 500 nmol L^–1^ for 5 min. The fluorescence imaging of fixated cells was performed using a CELENA S (LogosBio) fluorescence microscope, choosing the DAPI channel (excitation 375–328, emission 460–450) for the GQDs and the DSRed channel (excitation 530–520, emission 620–600) for PI stain.

## Results and Discussion

### Characterization of GQDs and GQD-GSH in Aqueous Dispersion

**Figure 1:**
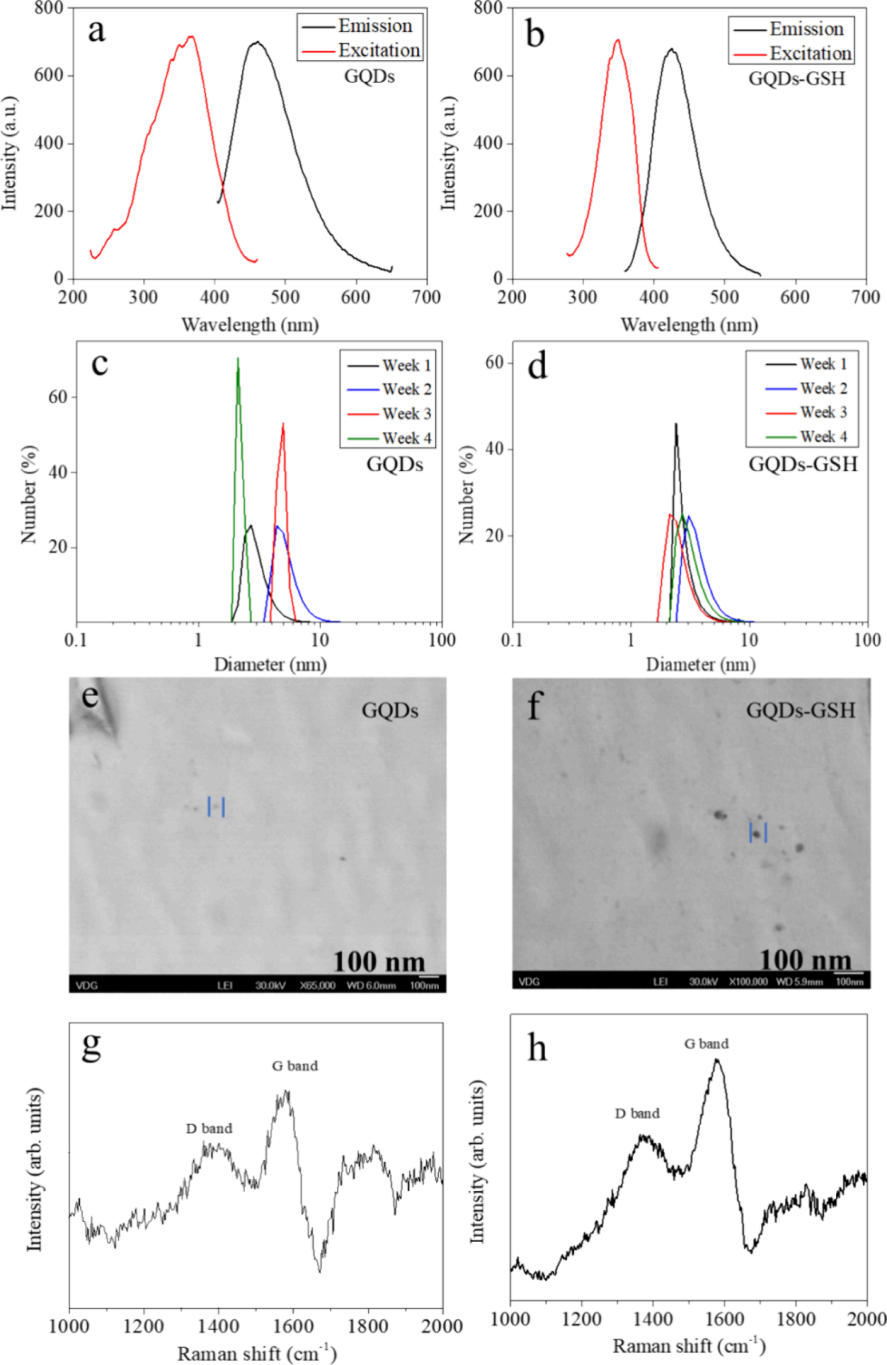
a) Photoluminescence spectrum emission and excitation GQDs in aqueous dispersion. b) Photoluminescence spectrum emission and excitation GQDs-GSH in aqueous dispersion. c) Hydrodynamic size distribution of GQDs in aqueous dispersion obtained by DLS. d) Hydrodynamic size distribution of GQDs-GSH in aqueous dispersion obtained by DLS. e) STEM image of GQDs. f). STEM image of GQDs-GSH. g) Raman spectra of GQDs. h) Raman spectra of GQDs-GSH.

The GQDs or GQDs-GSH aqueous dispersion were characterized using different techniques. The GQDs dispersion showed photoluminescence emission with a maximum at 458 nm (Figure 1a) when excited at 365 nm, indicating quantum confinement.^23,24^ Likewise, the GQDs-GSH presented maximum emission at 424 nm when excited at 347 nm (Figure 1b).

The total carbon content (TCC) in the original aqueous GQDs dispersions, prepared by hydro-exfoliation of 1 g of citric acid and after dialysis, required dilution to reduce TCC to about 52 μg L^–1^. The GQD-GSH amounted to 56 ng mL^–1^ after dialysis and dilution due to the higher intrinsic luminescence. The DLS technique was used to determine the hydrodynamic size of the nanomaterials and study their thermodynamic stability over 4 weeks. The GQDs average hydrodynamic size varied from 2.6 ± 0.6 nm to 9.9 ± 1.4 (Figure 1c) with no apparent tendency in function of time, which demonstrated a reasonable nanomaterial stability with some degree of heterogeneity in size but with no tendency to aggregation, probably due to the negative charge present on its surface. At pH 7.4, the carboxylic groups of the GQDs are deprotonated, affording a negatively charged surface (ζ-potential value of – 10.1 ± 0.6 mV).

For the GQDs-GSH, an average particle size was of the same order as the GQDs (varying from 5.4 ± 0.5 nm to 10.2 ± 2.7 nm), and no evident tendency growth was found in the function of time (weekly measurement) as seen in Figure 1d. Results indicated stability due to the surface charge (ζ-potential of – 5.7 ± 1.1 mV at pH 7.4) hindering aggregation.

Additionally, STEM images were acquired and presented in Figure 1e-f, which shows the structural attributes of GQDs and GQDs-GSH and provides insights into their morphology. The Raman spectra of GQDs and GQDs-GSH are shown in Figure 1**g-h**, exhibiting typical graphene D and G-bands at 1349 cm^–1^ and 1607 cm^–1^, respectively. Characteristic Raman features (bands D and G) indicated *sp*^2^ carbon in graphitic nanostructures.

The analysis of vibrational modes conducted through FTIR has provided valuable insights into the functional groups present in the synthesized nanomaterials. The frequency values, measured in cm^–1^, are systematically organized in Table S1. Precisely, the bands at 3490 cm^–1^ for GQDs and 3521 cm^–1^ for GQDs-GSH correspond to the asymmetric stretching of the O–H group (ν_O–H_). The asymmetric stretching mode of the C–H bond (ν_C–H_) is identified at 2946 cm^–1^ for GQDs and 2933 cm^–1^ for GQDs-GSH, indicating *sp*^2^-hybridized C atoms. Additionally, strong and broad bands at 1693 cm^–1^ for GQDs and 1697 cm^–1^ for GQDs-GSH correspond to the asymmetric stretching frequencies of C = O groups (ν_C=O_). These frequencies are associated with the νC = C stretching mode, which is distinctly observed at 1545 cm^–1^ for GQDs-GSH. This data confirms the presence of D and G bands in the Raman spectra, indicating the existence of *sp*^2^ C atoms within graphitic nanostructures.

The observed bands at 1402/894 cm^–1^ for GQDs and 1394/889 cm^–1^ for GQDs-GSH are attributed to angular bending and out-of-plane deformation of C–O–H subunits in carboxylic groups (COOH), respectively. The stretching bands associated with C–O groups are recorded at 1193 cm^–1^ for GQDs and 1172 cm^–1^ for GQDs-GSH. Notably, the nanomaterial functionalized with glutathione was anticipated to exhibit a weak band in the range of 2600–2550 cm^–1^, typically associated with thiol groups (S–H). However, no such bands were observed in this spectrum region (Figure S1). This absence implies that the S–H groups may have undergone oxidation to form S–S bridges during pyrolysis. An S–S vibration frequency substantiates this assertion at 486 cm^–1^, which is exclusively present in the GQDs-GSH spectrum, thereby confirming the successful functionalization of GQDs with glutathione.

### GQDs-Induced Antiproliferative Assay

The measured hydrodynamic diameter by DLS for GQDs of about 3–6 nm is compatible with cellular uptake. It is well-known that nanoparticles with particle sizes up to 150 nm can accumulate on cell tissue with further passive uptake through cell wall.^25^ Once inside the cell, the nanomaterial can affect cell division, leading to apoptosis depending on redox characteristics and the nature of surface chemistry.^26^

The cytotoxicity of the synthesized GQDs and their potential to inhibit cell viability were evaluated using the MTT assay, a widely established method for determining cell viability. The assay is based on mitochondrial dehydrogenases reducing MTT (a yellow tetrazole) to insoluble purple formazan crystals in metabolically active cells. The crystals are then solubilized, allowing their absorbance to be measured at 540 nm, directly correlating with the number of viable cells.

**Figure 2:**
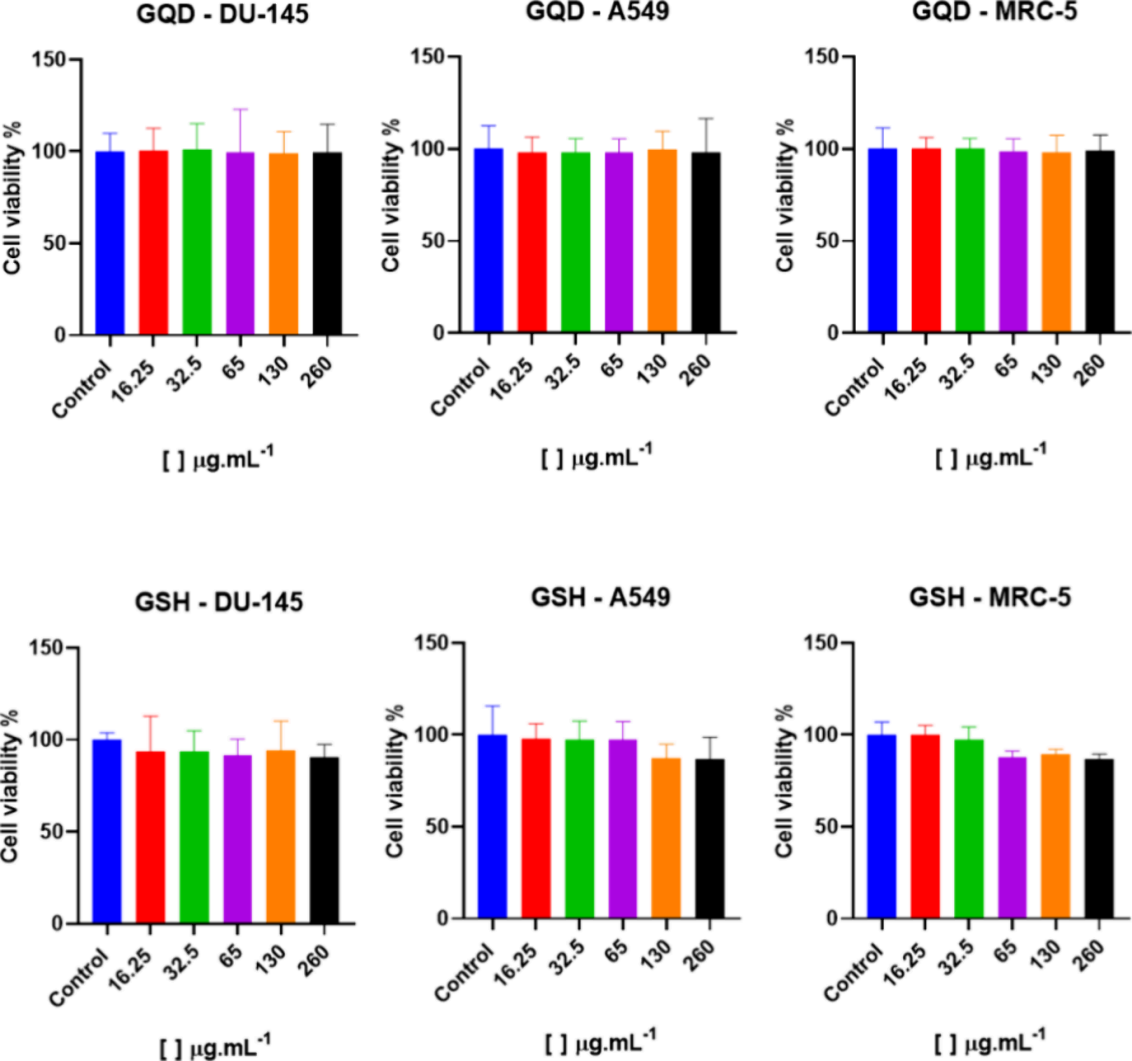
Cytotoxicity assessment of GQDs and GQDs-GSH was conducted at various concentrations (16.25 to 130 μg mL^–1^; *n* = 3) using A549 (lung cancer, ATCC – CCL-185), DU-145 (prostate cancer, ATCC – HTB-81), and MRC-5 (nontumor lung fibroblast, ATCC – CCL-171) cell lines, all maintained in DMEM supplemented with 10% heat-inactivated FBS.

This study investigated the cytotoxicity of GQDs on two human cancer cell lines, A549 (lung) and DU-145 (prostate), and on the nontumorigenic lung fibroblast cell line MRC-5. The nanomaterials’ dose, ranging from 16.25 to 260 μg mL^–1^ (based on TCC), was tested to evaluate potential dose-dependent effects. The results showed a biocompatibility trend with no significant reduction in cell viability compared to the negative control across the concentration range (Figure 2). The negative control, representing 100% cell viability, was established by adding ultrapure water to the cell medium.

Statistical analysis revealed no significant differences in cell viability at the *p* < 0.1 level, indicating that GQDs and their GSH-functionalized counterparts (GQDs-GSH) are nontoxic at these concentrations. These results suggest that both nanomaterials can be considered safe for further exploration in biomedical applications, particularly in contexts where biocompatibility is critical. This lack of cytotoxicity, even at higher concentrations, underscores the potential of GQDs as promising candidates for drug delivery, imaging, or other therapeutic applications.

### RBC Hemolytic Profile

Intravenous administration is usually used to improve the biodistribution of probes. Thus, hemolytic activity is a desired *in vitro* toxicity assessment for these biomaterials. This approach relies on measuring the extent of damage induced on red blood cell (RBC) membranes, quantifying the light extinction promoted by releasing hemoglobin at 540 nm^18^. Along these lines, the toxic effect on RBCs is ranked based on the observed hemolysis rate (%), and the following classification is used: 0–9% (nontoxic); 10–49% (slightly toxic); 50–89% (toxic); 90–100% (highly toxic).^27,28^ To establish a baseline for comparison, the Triton X-100 solution, at 1% (v/v), was used as a positive control, i.e., 100% of hemolysis, while PBS buffer (0.01 mol L^–1^; pH 4) was considered negative control, i.e., 0% of hemolysis. As a result, aliquots of 100 μL of the erythrocyte suspension and 100 μL of each GQD dispersion, at a concentration range of 13–112 μg mL^–1^, showed nontoxic to slightly toxic effects in erythrocyte hemolysis (below 12%). A summary of these data is presented in Table 1.

**Table 1:** Biochemical RBC Hemolysis Test in the Presence of Increasing Concentration of GQDs (13–104 μg mL^–1^) and GQDs-GSH (14–112 μg mL^–1^)^a^

**GQDs**		**GQDs-GSH**		**GQDs**	**GQDs-GSH**
**μg mL**^**–1**^	**extinction**	**μg mL**^**–1**^	**extinction**	**hem %**	
**104**	0.285 ± 0.062^b^	**112**	0.261 ± 0.081^b^	8.14 ± 0.15^b^	7.90 ± 4.99^b^
**52.0**	0.233 ± 0.026^b^	**56.0**	0.175 ± 0.026^b^	6.36 ± 0.74^b^	4.34 ± 1.35^b^
**26.0**	0.120 ± 0.006^b^	**28.0**	0.125 ± 0.006^b^	2.22 ± 0.41^b^	2.04 ± 2.32^b^
**13.0**	0.129 ± 0.016^b^	**14.0**	0.130 ± 0.016^b^	2.67 ± 1.34^b^	2.56 ± 0.31^b^
**(−)**	0.060 ± 0.002^b^	**(−)**	0.060 ± 0.002^b^	0.00	0.00
**(+)**	2.818 ± 0.699^b^	**(+)**	2.818 ± 0.699^b^	100	100

a The degree of damaged RBC was measured at 540 nm. The observed hemolysis rate is 0-9% non-toxic, 10-49% slightly toxic, 50-89% Toxic, and 90-100% highly toxic.(−) PBS buffer; (+) Triton X-100.

b Standard deviation.

### Hen’s Egg Test–Chorioallantoic Membrane (HET-CAM) Evaluation

**Figure 3:**
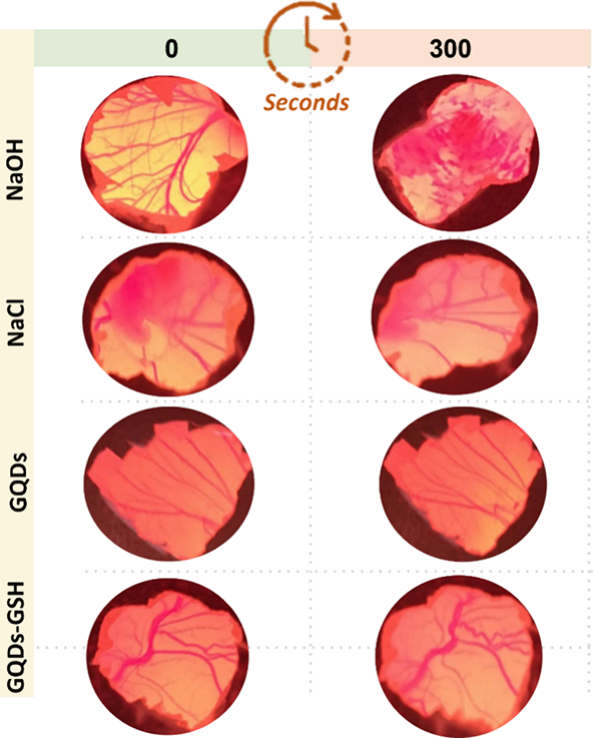
HET-CAM assay, representative images per group.

HET-CAM stands as a widely adopted method for evaluating skin and tissue irritation, serving as a substitute for conventional animal testing in safety assessments of novel extracts with potential biological applications.^29^ Applying 100 and 300 μL of a 0.1 mol L^–1^ sodium hydroxide (NaOH) solution to the intact CAM resulted in pronounced hemorrhage, subsequent lysis, and coagulation, exhibiting an escalating severity over the 300 s observation period (Figure 3). This pattern categorizes the NaOH solution as a potent irritant that causes significant tissue damage. In contrast, administration of the NaCl solution, matched in volume, onto the unaltered CAM manifested no perceptible visual reactions throughout the observation period (Figure 3). Interestingly, eggs treated with GQDs and GQDs-GSH exhibited a response profile comparable to that of the NaCl group, with the healthy CAM showing no observable visual changes over the 300 s duration (Figure 3).

The semiquantitative HET-CAM score elucidated the potency of the NaOH group at a concentration of 0.1 mol L^–1^, administered at a volume of 300 μL, in instigating a pronounced and widespread occurrence of hemorrhage, vascular lysis, and coagulation over a 300 s duration. In contrast, the NaCl group and GQD and GQD-GSH (at 100 and 300 μL) exhibited no reactions without visual alterations in the same exposure period (Table 2). Finally, eggs treated with the NaOH solution (0.1 mol L^–1^) showed a survival rate of 66% over the 600 s, while the NaCl group, GQDs, and GQDs-GSH exhibited no deaths. The HET-CAM data substantiate the viability of natural extracts as a biocompatible strategy for bioactive compounds. This assertion is supported by the markedly elevated irritation score observed in the GQDs and GQDs-GSH, starkly contrasting control groups that exhibited no discernible irritative or damaging effects on the healthy CAM tissue.

**Table 2:** Irritation Score and Irritancy Classification in the HET-CAM Assay^a^

group	irritation score	HET-CAM score
NaOH	19.13 ± 0.48	+++
NaCl	0.07 ± 0.00	–
GQDs	0.07 ± 0.00	–
GQDs-GSH	0.07 ± 0.00	–

a (−) No reaction, absence of hemorrhage, vascular lights, and coagulation over 300 s; (+) Slight reaction, isolated and slight hemorrhages, vascular lysis, and coagulations over 300 s; (+ + ) Moderate reaction, moderate hemorrhage, vascular lysis, and coagulations over 300 s; (+ + + ) Severe reaction, severe hemorrhage, vascular lysis, and coagulation over 300 s.^30,31^

### GQDs/DNA Binding Affinities

**Figure 4:**
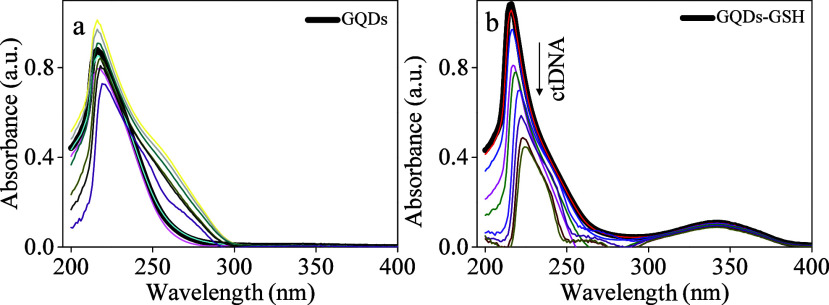
Absorption spectra of the (a) GQDs (149.5 mg L^–1^) in the absence and the presence of amounts of ctDNA (2.0 × 10^–6^ mol L^–1^ to 3.6 × 10^–5^ mol L^–1^). (b) GQDs-GSH (102.9 mg L^–1^) in the absence and the presence of quantities of ctDNA (2.6 × 10^–6^ mol L^–1^ to 4.4 × 10^–5^ mol L^–1^). The study was performed at room temperature in Tris–HCl/NaCl buffer (pH 7.4).

UV–visible absorption spectroscopy and circular dichroism (CD) are usually combined to investigate different modes of interactions between DNA and potential binders, as each DNA-ligand complex reveals specific spectral behavior. At the molecular level, GQDs-based materials have essential planar geometry. Thus, it would be expected that GQDs afford some DNA structural alterations by intercalation binding mode.^32−35^ Here, GQDs and GQDs-GSH binding properties to ctDNA were first monitored employing absorption UV–visible spectroscopy. Accordingly, under the increasing concentration of ctDNA, 0.26×10^–5^ – 4.4×10^–5^ mol L^–1^, the GQDs-GSH electronic spectrum with maximum absorbance at 218 nm exhibit significative hypochromic effect and slight bathochromism as seen in Figure 4b. This behavior suggests a good affinity for forming a binary GQDs-GSH/DNA complex (*K*_*b*_ = 1.80×10^4^). This behavior is corroborated by one absorption band observed at 340 nm, ascribed to the n→π* electronic transition, indicating that the *N*-doped surface of GQDs-GSH is involved with noncovalent contacts into the binding site. Nonetheless, the GQDs electronic spectrum (Figure 4a) implies no good affinity by ctDNA structure as a random variation of intensities appears under increasing biomolecule concentration. Also, a similar n→π* electronic transition band was not found in the GQDs electronic spectrum.

**Figure 5:**
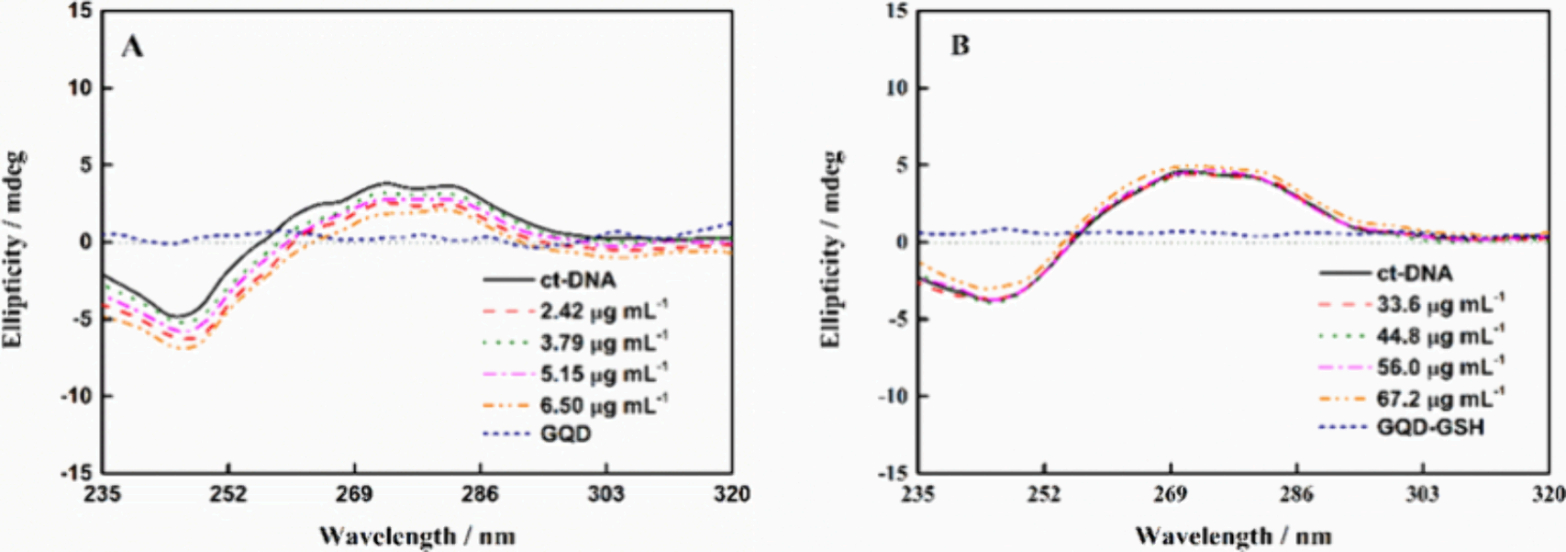
Circular dichroism spectra for ctDNA with increasing concentration of (**A**) GQDs (0–6.50 μg mL^–1^) and (**B**) GQDs-GSH (0–67.2 μg mL^–1^). Also, both GQDs and GQDs-GSH CD spectra without the addition of ctDNA were monitored, and they have been shown as navy short dash lines.

In Figure 5, the CD spectra of ctDNA in the absence of nanomaterials exhibit distinctive positive (275 nm) and negative (245 nm) bands associated with the base pair π-π* stacking interaction, and the right-handed conformation of ctDNA (B-form), respectively. Notably, the increased concentration of either GQDs or GQDs-GSH does not induce any discernible alteration in the ctDNA helical form. This observation is consistent with weak noncovalent interactions occurring with the double-stranded structure. In addition, it rules out an intercalative binding mode, which could lead to distortion of the secondary DNA structure, resulting in the loss of the observed CD spectrum.

### HSA Binding Study

**Figure 6:**
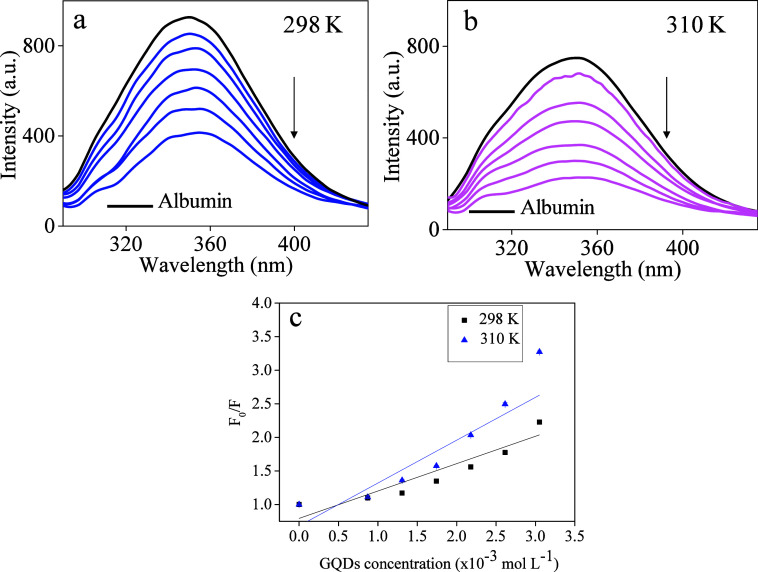
Fluorescence emission spectra (347 nm) for the HSA solution (0.4 μmol L^–1^) and with increased amounts of GQDs (10.5 to 36.6 mg L^–1^) in Tris-HCl buffer pH 7.4. (a) 298 K (b) 310 K (c) Fluorescence quenching correlation in the two temperatures.

**Figure 7:**
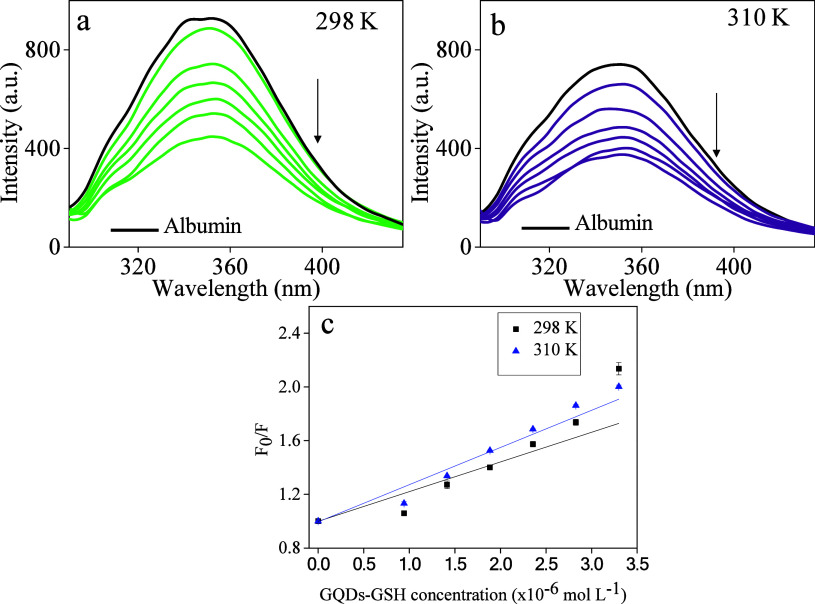
Fluorescence emission spectra (347 nm) for the HSA solution (0.4 μmol L^–1^) and with increased amounts of GQDs-GSH (0.011 to 0.039 mg L^–1^) in Tris-HCl buffer pH 7.4. (a) 298 K (b) 310 K (c) Fluorescence quenching correlation in the two temperatures.

HSA exhibits intrinsic fluorescence with an emission maximum of 347 nm (excitation at 280 nm),^18,36,37^ which is very sensitive and easily affected by other chemical species.^22^ The addition of increasing amounts of GQDs (10.5 to 36.6 mg L^–1^) and GQDs-GSH (0.011 to 0.039 mg L^–1^), as shown in Figures 6**a-b** and 7**a-b**, respectively, resulted in a quenching of the fluorescence intensity of the protein in experiments conducted at two different temperatures (298 and 310 K). The temperature of 310 K was chosen to simulate the human body, while 298 K is the room temperature.

Since GQDs are not discrete molecules, they do not have precise molar concentrations. To estimate the molar masses, we applied a 12 g mol^–1^ value for carbon based on the TCC values. This approach allows for a more accurate comparison between the nanoparticles. It was observed that significantly fewer molar amounts of GQDs-GSH were needed to achieve the same level of quenching as GQDs. This result indicates that sulfur (S) and nitrogen (N) modifications on the nanoparticles enhance the effectiveness of GQDs-GSH in interacting with biomolecules, particularly HSA.

The intermolecular interactions between HSA and GQDs-GSH were further investigated to elucidate the underlying mechanisms. Experiments conducted at controlled temperatures are useful for clarifying the nature of the interaction between HSA and GQDs-GSH, as the dependence of the luminescence quenching mechanism on temperature reveals its nature, which can be static, dynamic, or a combination of both processes. As seen in Figure 6c, the fluorescence quenching induced by GQDs-GSH becomes more pronounced at higher temperatures, as evidenced by the significantly higher sensitivity of the linear fit of the normalized HSA fluorescence curve in relation to the quencher (GQDs-GSH) at 310 K compared to the one obtained at 298 K. However, the overall data showed a tendency to produce a curvature (a quadratic profile) common when a mixed process comprising both the long-term interaction between nanomaterial and protein (static quenching) and collisions between these entities with radiationless deactivation of HSA during the excited state lifetime (dynamic quenching). This partial static behavior implies that GQDs-GSH can exhibit favorable biodistribution in biofase as HSA has a pivotal role as the most abundant plasma protein, being a primary modulator of fluid distribution between body compartments and a carrier for numerous endo- and exogenous compounds.^36−40^

**Figure 8:**
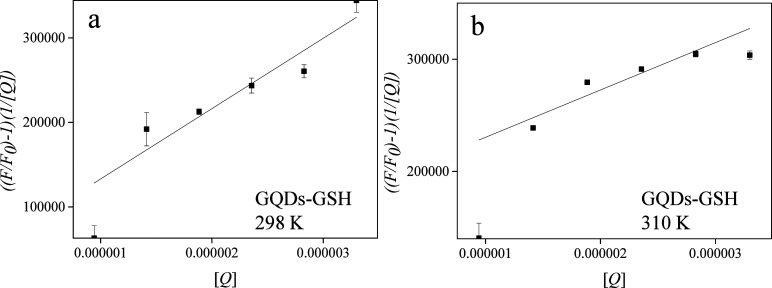
Apparent constant (*K*_*ap*_) plots concerning the mixed quenching process caused by GQDs-GSH (0.011 to 0.039 mg L^–1^), [Q], in the HSA (0.4 μmol L^–1^) fluorescence (measured at 347 nm) in Tris-HCl buffer pH 7.4. (a) 298 K (Y = (8.3 ± 1.8) × 10^10^ X + (0,5 ± 0.4 × 10^5^) (b) 310 K (Y = (4.2 ± 1.0) × 10^10^ X + (1.8 ± 0.2 × 10^5^).

As follows, our results indicate that GQDs-GSH exhibit a mixed quenching behavior, whereas pure GQDs predominantly undergo static quenching. Thus, temperature-dependent fluorescence experiments and mathematical modeling were employed herein to deconvolute the contributions of static (*K*_*S*_) and dynamic (*K*_*D*_) quenching. A mathematical approach was employed to assess the relative importance of each process. To achieve this, the apparent quenching constant (*K*_*ap*_) was determined by plotting the product of the normalized fluorescence ((F_0_/F) − 1) and the inverse of the quencher concentration (GQDs-GSH) as a function of the quencher concentration as indicated in eq 6 and in Figure 8. The sensitivity (*K*_*D*_*K*_*s*_) and the intersection (*K*_*D*_ + *K*_*s*_) of the curve with linear behavior can be determined from this equation. From these two mathematical relations, *K*_*D*_ and *K*_*S*_ values can be estimated **(**Table 3).
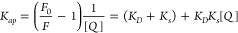
6

**Table 3:** Static Quenching Constants (*K*_*S*_), Dynamic Quenching Constants (*K*_*D*_), Apparent Binding Constant (*K*_*b*_), and the Number of Binding Sites (*n*) regarding the Interaction of GQDs-GSH with HSA

T (K)	***K*_*S*_**	***K*_*D*_**	*K*_*b*_**(mol L**^**–1**^**)**	***n***
298	2.7 × 10^5^	3.0 × 10^5^	1.7 × 10^9^	1.7
310	1.3 × 10^5^	3.2 × 10^5^	1.8 × 10^7^	1.3

Both constants exhibit a similar magnitude, indicating a mixed behavior of the system. Additionally, it is significant that an increase in temperature (from 298 to 310 K) leads to an elevation in the *K*_*D*_ value during the dynamic suppression process, possibly due to the increased kinetic energy of the molecule. Conversely, the constant *K*_*s*_ decreases with rising temperature, hindering the long-term interaction between HSA and GQDs-GSH. Consequently, the temperature-dependent fluorescence quenching experiments, in conjunction with mathematical modeling, demonstrate that, in contrast to GQDs, dynamic quenching exhibits an increase with temperature, whereas static quenching shows a decrease. This behavior indicates that the N and S functionalities present in GQDs-GSH enhance their interaction with the HSA protein through a dynamic process. Therefore, it is confirmed that GSH functionalization significantly alters the interaction profile of these nanomaterials with biomolecules, leading to distinct quenching behaviors.

It is essential to recognize that measurements of quantum yield and fluorescence lifetime can enhance this analysis by utilizing a time-resolved fluorescence lifetime apparatus. Nonetheless, the methodologies employed herein offer substantial evidence regarding the nature of interactions and the impact of surface functionalization on quenching mechanisms.

The apparent binding constant (*K*_*b*_) and the number of binding sites (*n*) for the interaction of GQDs-GSH with HSA were estimated using eq 4 and are presented in Table 3. In this calculation, the value of F (fluorescence in the presence of GQDs-GSH) is considered to represent total quenching, regardless of the process involved (static or dynamic), based on the original experimental data.

Thermodynamic data were calculated and placed in Table 4. The enthalpy variation for the formation of a GQDs-GSH/HSA complex was estimated as −46.7 kJ mol^–1^ by using the *K*_*S*_ values, calculated at 298 and 310 K in the van’t Hoff eq (eq 7) with *R* as the gas constant (8.314 J mol^–1^ K^–1^). From *K*_*b*_ values, the Gibbs free energy (ΔG°) values were calculated (eq 8), showing spontaneity in the formation of the GQDs-GSH/HSA complex (ΔG° < 0) and a slight decrease (variation of about 10 kJ mol^–1^) in ΔG° when the temperature was elevated. Finally, entropy variation (ΔS°), at 298 K, was calculated using (eq 9), and a negative value (−320 J K^–1^ mol^–1^) was obtained. As the ΔH and ΔS values were both negative, it is suggested that hydrogen bonds with (or not) van der Waals interactions are predominant in the interaction between GQDs-GSH and HSA.
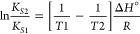
7
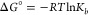
8
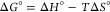
9

**Table 4:** Thermodynamic Data for the GQDs-GSH/HSA System^a^

T (K)	**ΔH°(kJ mol**^**–1**^**)**	**ΔS°(J mol**^**–1**^ **K)**	**ΔG°(kJ mol**^**–1**^**)**
298	–46.7	–320	–52.6
310	–46.7	n.d.	–43.0

a n.d. = not determined.

Recent studies have shown that GQDs-GSH can serve as turn-off probes to detect biomolecules and bioactive compounds, as demonstrated through investigations into their interactions with glutathione and specific proteins.^41,42^ Unlike these methods, our research revealed that HSA has the potential to transport GQDs-GSH in biological phases by examining the formation of potential complexes with this biomolecule. Additionally, these findings are consistent with recent research from our group, which observed that N-doped GQDs (GQD-U, produced with urea as the N source) can detect HSA in physiologically mimetic conditions.^43^

### Apoptotic Cell Staining Assay by Fluorescence Microscopy

Apoptotic cell staining assays using fluorescence microscopy are essential in biomedical research and clinical diagnostics to detect and visualize apoptotic cells. These assays provide insights into apoptosis by highlighting cellular features such as nuclear condensation and DNA fragmentation. Following 24 h of incubation, 1 μL of cisplatin (6.25 × 10^–6^ and 12.5 × 10^–6^ mol L^–1^) was added to the wells. Then, dispersions of GQDs and GQDs-GSH were introduced to the cells and incubated for another 48 h.^44^

**Figure 9:**
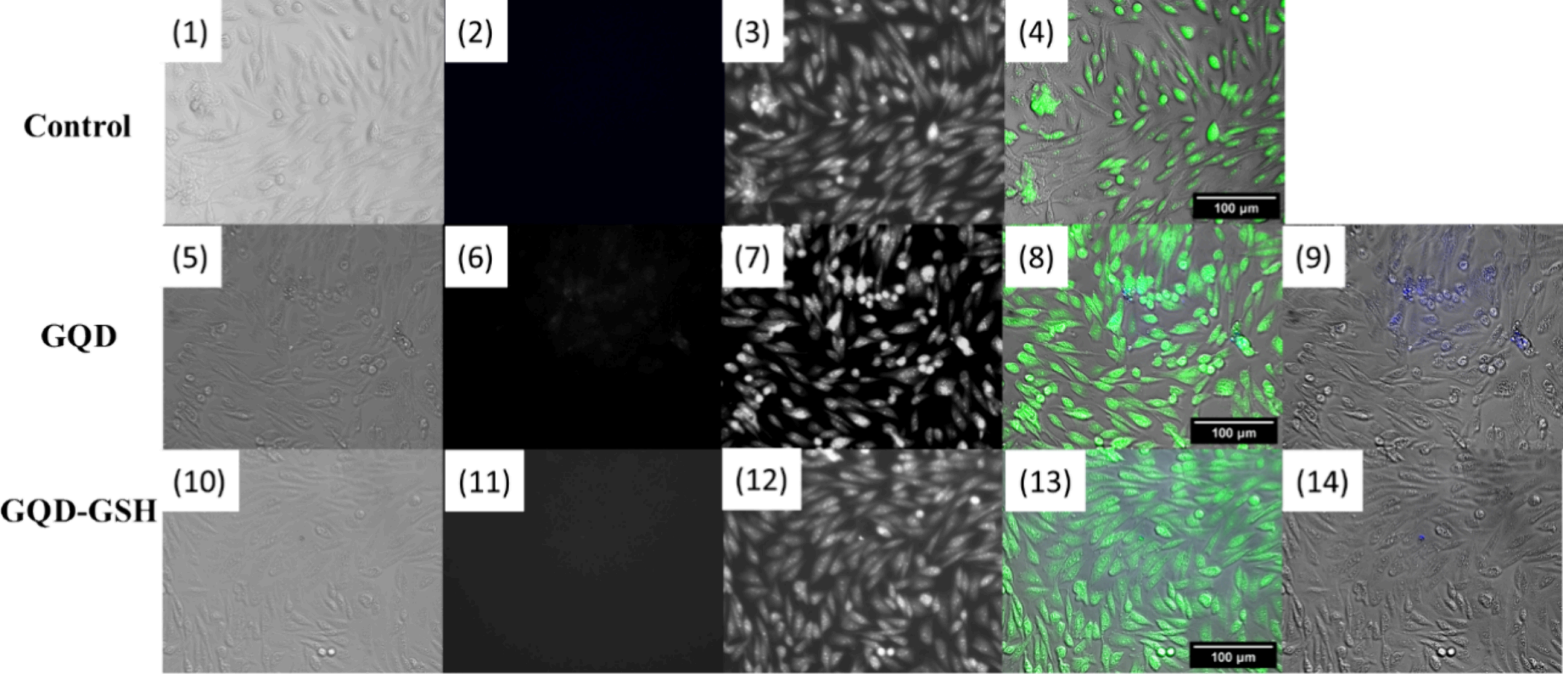
Fluorescence microscopy images capture the cultured MRC-5 cell line under various conditions, specifically in the absence or presence of GQDs and GQDs-GSH at a concentration of 100 μg, along with exposure to the apoptotic agent cisplatin. The DAPI channel (excitation 375/28 nm, emission 460/50 nm) was chosen to visualize the GQDs. In comparison, the EYFP channel (excitation 500/20 nm, emission 535/30 nm) was used for the CellMask Green plasma membrane staining to visualize all cells. In the figure, numbers 1, 5, and 10 indicate transmitted light images; numbers 2, 6, and 11 correspond to images captured with the DAPI filter; numbers 3, 7, and 12 represent images captured with the EYFP filter; numbers 4, 8, and 13 show merged images; and numbers 9 and 14 show the merging of transmitted light with the DAPI filter.

Fluorescence microscopy, utilizing the DAPI channel to capture images near the emission region of both GQDs, revealed that these nanomaterials were present in areas containing apoptotic bodies (Figure 9**(9)**) and near the membranes of spheroid-shaped cells (Figure 9**(14)**). These observations might indicate a potential interaction of GQDs and GQDs-GSH with apoptotic structures, however, the qualitative nature of the analysis does not allow for a definitive claim of specific selectivity toward apoptotic cells. Since apoptotic cells have compromised membranes, their increased permeability may facilitate the internalization of nanomaterials, regardless of specific interactions. Therefore, further studies are required to determine the differential selectivity of these nanoparticles.

## Conclusion

In summary, this study systematically investigated the physicochemical characteristics of GQDs, and an *N*-doped variant modified with glutathione, GQDs-GSH, providing preliminary insights into their potential biomedical applications. GQDs-GSH exhibited heightened stability and reduced aggregation, which were attributed to *N*-functionalization. STEM imaging confirmed the morphology of both GQDs, while Raman spectra indicated the *sp*^2^ carbon hybridization typical of graphitic nanostructures. Biological assessments revealed the biocompatibility of GQDs, establishing their suitability for biological applications. The antiproliferative assay demonstrated their safety, while the RBC hemolysis and HET-CAM experiments corroborated their low toxicity and nonirritating nature to mucosal tissues, respectively.

Examining biomolecular interactions, GQDs-GSH demonstrated better binding affinity to ctDNA compared to GQDs. In HSA binding studies, GQDs exhibited dynamic quenching, while GQDs-GSH displayed a combination of static and dynamic mechanisms, underscoring the influence of glutathione modification on the biological properties.

Further research is required to develop a nanomaterial with selective properties; however, this study constitutes an initial step toward understanding the system’s behavior. The apoptotic cell staining conducted through fluorescence microscopy provided qualitative observations: GQDs appeared to accumulate in regions with apoptotic bodies, whereas GQDs-GSH tended to localize near the membranes of spheroid-shaped cells. Given that apoptotic cells exhibit increased membrane permeability, these findings suggest that nanomaterial uptake may be influenced by this characteristic rather than selective adherence. Future investigations employing quantitative fluorescence analysis and complementary techniques such as flow cytometry are necessary to further elucidate the potential selectivity of these nanomaterials toward apoptotic cells.

## Supporting Information Available

**Figure S1:** FTIR plots about the frequency bands associated with the active vibrational modes for GQDs and GQDs-GSH. **Table S1**: Summary of selected frequencies for the active vibrational modes of GQDs and GQDs-GSH (PDF)
