# Supplementary material for: InVitro and Ex Vivo Biocompatibility, Biomolecular Interactions, and Characterization of Graphene Quantum Dots and Its Glutathione-Modified Variant for Qualitative Cell Imaging
Source: ACS Omega. 2025 Apr 15;10(16):16194–206. doi: 10.1021/acsomega.4c10014 (PMC12044483; doi:10.1021/acsomega.4c10014)
Supplement: Supplementary file 1 — ao4c10014_si_001.pdf [file ao4c10014_si_001.pdf]

In vitro and *ex vivo* biocompatibility, biomolecular interactions, and characterization of graphene quantum dots and its glutathione-modified variant for qualitative cell imaging

Marlin Pedrozo-Peñafiel[a], Luis Gutierrez-Beleño[a], Cesar A. D. Mendoza[b,c], Fernando L. Freire-Júnior[c], Mauro A. Lima[d], Tamara Teixeira[d], Fillipe V. Rocha[d], Gabriela V. S. Zolin[e], Saulo S. Garrido[e], Ana B. Lazzarini[f], Adelino V. G. Netto[f], Felipe F. Haddad[g], Emilio E. João[g], Jean L. Santos[g], Cauê B. Scarim[g], Renan L. Farias[a], and Ricardo Q. Aucélio[a]*

[a]*Dep. of Chemistry, Pontifical Catholic University of Rio de Janeiro (PUC-Rio), Rio de Janeiro, RJ, 22451-900, Brazil*

[b]*Dep. of Electric Engineering, Rio de Janeiro State University (UERJ), Rio de Janeiro, RJ, 20550-900, Brazil*

[c]*Dep. of Physics, Pontifical Catholic University of Rio de Janeiro (PUC-Rio), Rio de Janeiro, RJ, 22451-900, Brazil*

[d]*Dep. of Chemistry, Federal University of Sao Carlos (UFSCar), Sao Carlos, SP, 13565-905, Brazil*

[e]*Dep. of Biochemistry and Organic Chemistry, Sao Paulo State University (Unesp), Araraquara, SP, 14800-060, Brazil*

[f]*Dep. of Analytical, Physicochemical and Inorganic Chemistry, Sao Paulo State University (Unesp), Araraquara, SP, 14800-060, Brazil*

[g]*School of Pharmaceutical Sciences, Sao Paulo State University (Unesp), Araraquara, SP, 14800-903, Brazil*

* Corresponding author: Renan Lira de Farias (renan.farias@puc-rio.br);

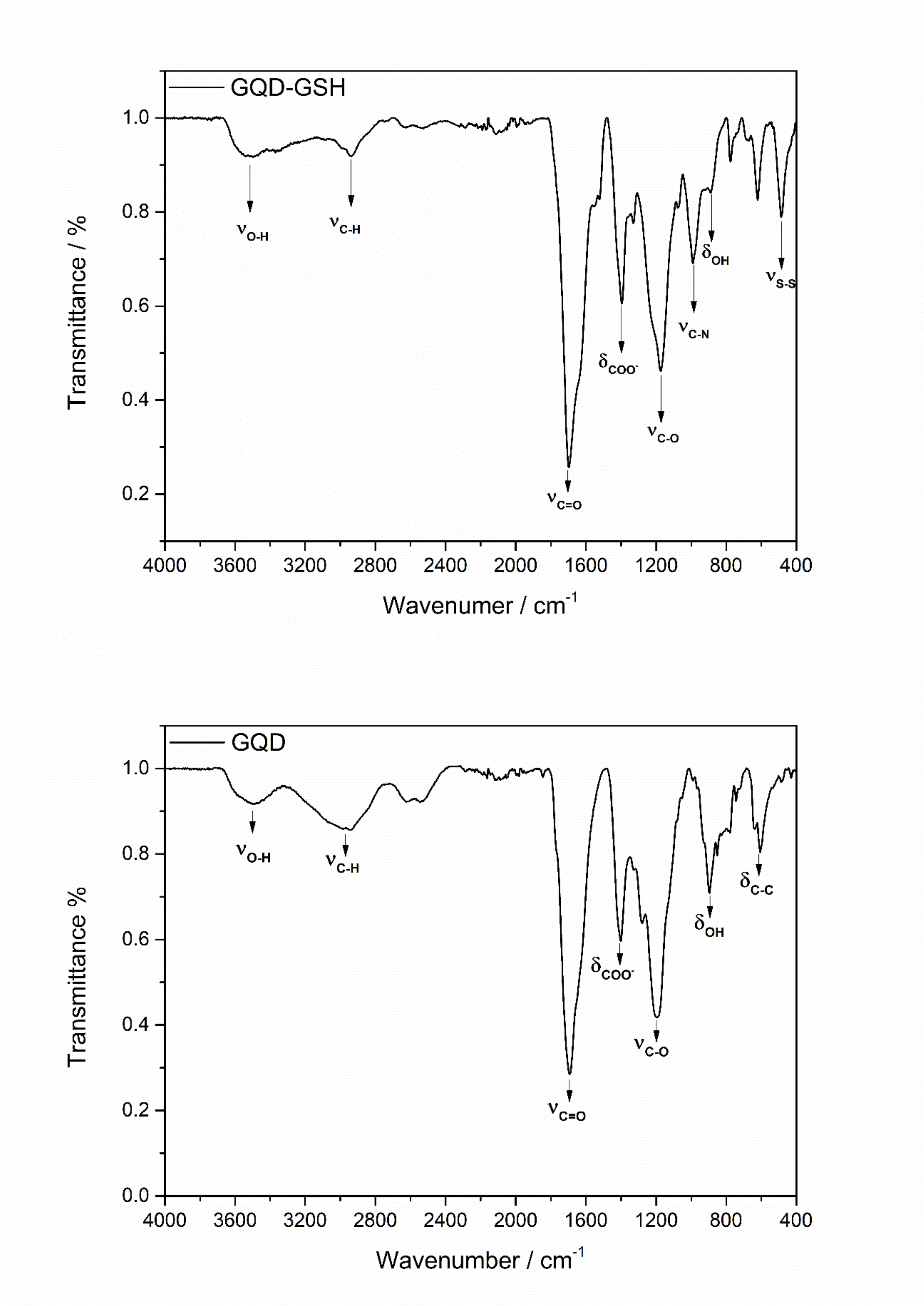

Figure S1. Frequency bands associated with the active vibrational modes for GQDs and GQDs-GSH. These data were collected using a Bruker ALPHA II FTIR spectrometer with an Eco-ATR QuickSnap Sampling Module (ZnSe crystal).

Table S1. Summary of selected frequencies for the active vibrational modes of GQDs and GQDs-GSH.

**Assignments** [1]	**GQDs**	**GQDs-GSH**
	**cm^{-1}**	
$\nu_{O\text{-}H}$	3490	3521
$\nu_{C\text{-}H}$	2946	2933
$\nu_{C=O} + \nu_{C=C}$	1693	1697
ν_{COO^-}	1402	1394
$\nu_{C\text{-}O}$	1193	1172
$\delta_{C\text{-}N}$	-	989
$\delta_{O\text{-}H}$	894	889
$\delta_{S\text{-}S}$ [2]	-	485

References

[1] Shi, W.; Fan, H.; Ai, S.; Zhu, L. Preparation of fluorescent graphene quantum dots from humic acid for bioimaging application. *The Royal Society of Chemistry* **2015** 39 (9) 7054-7059. DOI: 10.1039/C5NJ00760G

[2] Constance E. N. et al., Effect of Microwave Heating on the Crystallization of Glutathione Tripeptide on Silver Nanoparticle Films. *The Journal of Physical Chemistry C* **2017** *121* (10), 5585-5593. DOI: 10.1021/acs.jpcc.6b11952
